# Frosted branch angiitis after smallpox vaccination

**DOI:** 10.1016/j.ajoc.2022.101622

**Published:** 2022-07-09

**Authors:** Kristen Collister, Sam S. Dahr

**Affiliations:** a Dean McGee Eye Institute, Oklahoma University Health Science Center, Oklahoma City, OK, USA

**Keywords:** Smallpox vaccine, Retinal vasculitis, Frosted branch angiitis, Uveitis, Inflammation

## Abstract

**Purpose:**

To describe a patient presenting with frosted branch angiitis soon after small-pox vaccination.

**Observations:**

Frosted branch angiitis (FBA) is an acute onset retinal vasculitis featuring prominent perivascular sheathing in otherwise healthy individuals. FBA has been associated with noninfectious and infectious etiologies. This report describes a twenty-year-old African American female who developed bilateral frosted branch angiitis one week after small-pox vaccination. At presentation, the patient had bilateral, para-central visual field defects and subjective visual disturbances. On dilated exam, the patient demonstrated diffuse vasculitis bilaterally. The patient's field defects and clinical exam responded dramatically to oral prednisone therapy.

**Conclusions and Importance:**

Acute idiopathic frosted branch angiitis is a rare condition which was temporally associated with small-pox vaccination.

## 1 Introduction

Frosted branch angiitis (FBA) is the clinical appearance of sheathing of retinal vessels, resembling the frost on a tree branch.^1^ FBA has been associated with various etiologies including leukemia & lymphoma,^2^ auto-immune conditions (e.g. Behcets, Crohn's^3^^,^^4^), herpes simplex virus,^5^ varicella zoster virus,^6^ and toxoplasmosis.^7^^,^^8^ While some patients have systemic symptoms, other young, healthy individuals lack systemic findings.^9^ This case report describes a young female patient with idiopathic frosted branch angiitis and is the only report in the literature of FBA temporally associated with small-pox vaccination.

## 2 Case report

**Fig. 1:**
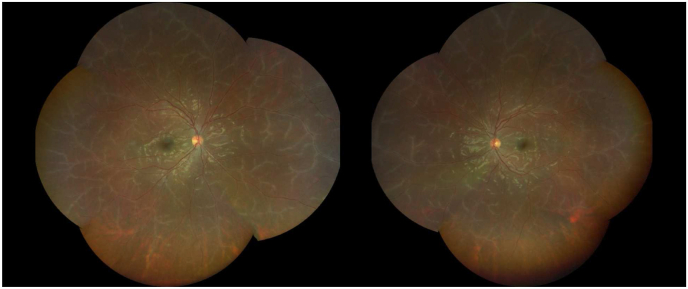
Dilated fundus examination showed profound vascular sheathing bilaterally, predominantly of veins but with some arteriolar involvement.

**Fig. 2:**
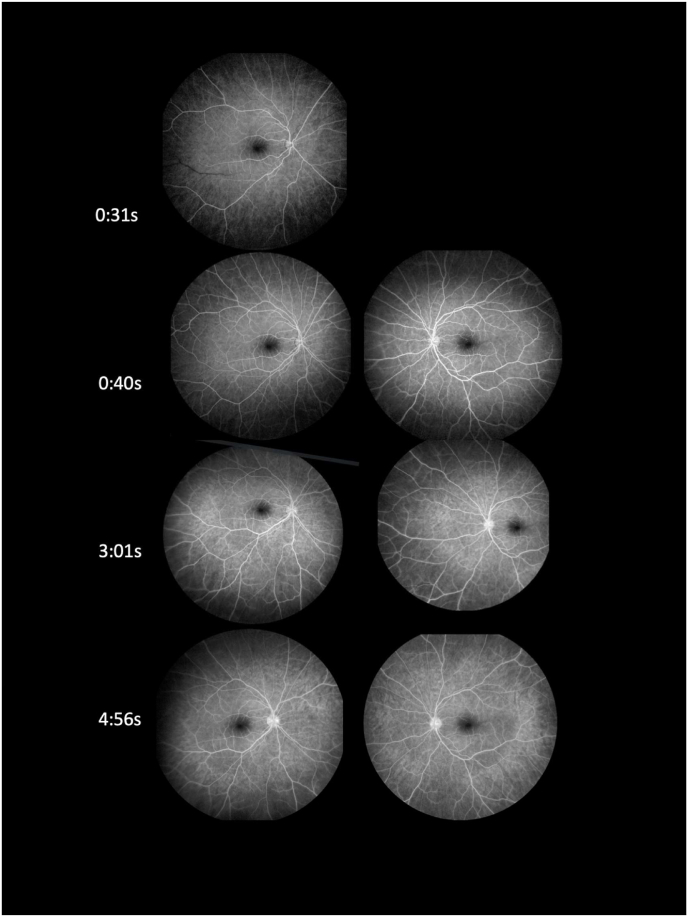
Fluorescein angiogram on presentation demonstrated diffuse vascular sheathing bilaterally without leakage.

A twenty-year-old African American female presented to the Dean McGee Eye Institute complaining of 3–4 days of “static” in her vision. Past medical history was unremarkable. The patient was active duty in the military. Review of systems was negative except for mild, intermittent headaches. On further questioning, she recalled receiving a small-pox vaccination (ACAM2000) 10–11 days prior to presentation. Visual acuity was 20/15 in each eye. External exam was unremarkable. Slit lamp exam of the anterior segment showed half plus diffuse conjunctival hyperemia bilaterally but was otherwise unremarkable. Dilated fundus examination showed profound vascular sheathing bilaterally, predominantly of veins but with some arteriolar involvement (Fig. 1). Visual fields revealed a para-central defect bilaterally and OCT demonstrated corresponding macular edema. Fluorescein angiogram (Fig. 2) showed an absence of leakage from the affected blood vessels. Laboratory testing including ACE, ANCA, QuantiFERON Gamma, HIV rapid test, VDRL, RPR, FTA-ABS, CBC, BMP, ESR, and CRP, was subsequently negative or normal. ANA was positive. MRI of the brain was unremarkable. Empiric oral prednisone at a dose of 60 mg was initiated.

At two-week follow-up, all visual symptoms had resolved. Visual field and OCT showed resolution of visual field defects and associated macular edema respectively. Fundus examination showed near complete resolution of the vascular sheathing. The patient's oral prednisone was subsequently tapered over four weeks, at which the conclusion she had no recurrence.

## 3 Discussion

The clinical presentation of frosted branch angiitis does not correlate with any single etiology. Rather FBA can be considered a clinical sign. As such, we considered numerous etiologies in our differential diagnosis. The patient's laboratory testing was not consistent with syphilitic nor tubercular infection. The bilaterality and symmetry of involvement suggested against herpes simplex virus or varicella zoster virus. While the ANA was qualitatively positive, an analysis of data from the National Health and Nutrition Examination Survey shows a significant proportion of the U.S. population has antinuclear antibodies.^10^ This patient had no other systemic, ophthalmic (cotton wool spots, hemorrhage, ischemia, etc.) or laboratory findings to suggest lupus. There were no history or laboratory findings to suggest leukemia or lymphoma. MRI of the brain did not show any demyelinating findings to suggest multiple sclerosis. This patient did not show leakage of dye from affected retinal vessels. The angiographic findings of FBA may vary and may not feature prominent leakage.^11^ In this patient, the absence of leakage suggests perivascular infiltration *without* breakdown of endothelial cell tight junctions. The relatively early presentation of the patient post-vaccination may explain the lack of leakage. Alternatively, the pathogenesis of this specific and presumably post-vaccine FBA may somehow spare the endothelium.

We identified the smallpox vaccination as the probable etiology of this patient's frosted branch angiitis. The smallpox vaccination was first developed in the 1798 by Edward Jenner. Subsequently, the vaccine was used to eradicate the smallpox virus in the 1970's. Due to recent concern for bio-terrorism, military groups throughout the world have begun to routinely vaccinate all service members. The vaccine is a live vaccina virus. Therefore, it induces both antibody and cell-mediated immunity.^12^ There are two types of vaccines which are currently on the market ACAM2000, and JYNNEOSTM (also known as Imvamune or Imvanex).^12^ The ACAM2000 has been used extensively by the military since 2007 and was the vaccine administered to this patient. ACAM2000 is a replication-competent vaccinia virus that can be transmitted from the vaccinated individual to others. In contrast, JYNNEOSTM is a replication-incompetent vaccine and cannot transmit from the vaccinated individual; therefore it can be used in the immunocompromised.^12^

All live, replication-competent versions of the vaccine (including the ACAM2000) carry significant risks of side effects. These include dermatologic, cardiac, immunologic, developmental, and ocular conditions. The inoculation site produces a large pustule. This pustule can be a source for auto-inoculation or bacterial superinfection. Vaccine recipients can also develop a general vaccina in which a disseminated vesicular rash occurs. Those with pre-existing atopy are at high risk to develop eczema vaccina – a serious complication which includes fever, lymphadenopathy and possible death. Other rare side effects include fetal vaccina, postvaccinal encephalitis, myocarditis/pericarditis, and dilated cardiomyopathy.^13^

Replication-competent vaccines can also cause numerous ocular side effects. Smallpox vaccination has been associated with blepharitis, conjunctivitis, and keratitis, likely related to an auto-inoculation mechanism where patients touch the vaccine injection site and subsequently touch the eyelids. Smallpox infection has been reported to cause iritis, iridocyclitis, retinitis, chorioretinitis, or optic neuritis.^14^ The only reports of smallpox vaccination associated ocular side effects are through the Vaccine Adverse Event Report System (VAERS). There was one report of optic neuritis 14 days after DryVax Immunization and two self-reported cases of uveitis (one unspecified and the other anterior uveitis) following smallpox vaccination of unknown type.^15^

Vaccinations other than smallpox have been implicated in uveitis, interstitial keratitis and optic neuritis. These reactions may be an immunologic response to the vaccine or its adjuvants.^16^ Several proposed mechanisms for retinal vasculitis secondary to vaccination include: immune complex mediated damage to the vessel wall, activation of B and/or T cells through molecular mimicry, and direct microbial invasion into endothelial cells.^17^ A case report from Scotland described two patients who developed arteriolar vasculitis following vaccination. The first patient presented with cotton wool spots, splinter hemorrhages and peripapillary arteriolar vasculitis after swine-flu immunization. The second patient presented after receiving three vaccinations: diphtheria, tetanus, and polio, hepatitis A, and typhoid. This individual developed unilateral optic disc swelling, submacular fluid and sheathing of arterioles.^18^ Viral vaccines, particularly influenza and hepatitis B, are also known to have a higher incidence of vasculitic reactions.^19^

In this report, the patient's unremarkable medical history and laboratory workup combined with the close temporal relationship to the vaccination event indicates smallpox vaccination was the likely etiology of the acute idiopathic frosted branch angiitis. Oral corticosteroid therapy may hasten resolution of anatomic and associated functional abnormalities.

## 4 Conclusions

Frosted branch angiitis is a rare entity that has a broad differential diagnosis. Our patient presented with acute idiopathic frosted branch angiitis shortly after smallpox vaccination. Ultimately, she had an excellent response to steroids. To our knowledge, this is the first case report of frosted branch angiitis temporally associated with smallpox vaccination.

## Patient consent

The patient provided written consent to publication of the case.

## Funding

No funding or grant support.

The following authors have no financial disclosures: Dr. Kristen Collister and Dr. Sam S. Dahr.

## Authorship

All authors attest that they meet the current ICMJE criteria for Authorship.

## Intellectual property

We confirm that we have given due consideration to the protection of intellectual property associated with this work and that there are no impediments to publication, including the timing of publication, with respect to intellectual property. In so doing we confirm that we have followed the regulations of our institutions concerning intellectual property.

## Research ethics

We further confirm that any aspect of the work covered in this manuscript that has involved human patients has been conducted with the ethical approval of all relevant bodies and that such approvals are acknowledged within the manuscript.

IRB approval was obtained (required for studies and series of 3 or more cases).

Written consent to publish potentially identifying information, such as details or the case and photographs, was obtained from the patient(s) or their legal guardian(s).

## Authorship

The International Committee of Medical Journal Editors (ICMJE) recommends that authorship be based on the following four criteria:

1. Substantial contributions to the conception or design of the work; or the acquisition, analysis, or interpretation of data for the work; AND
2. Drafting the work or revising it critically for important intellectual content; AND
3. Final approval of the version to be published; AND
4. Agreement to be accountable for all aspects of the work in ensuring that questions related to the accuracy or integrity of any part of the work are appropriately investigated and resolved.

All those designated as authors should meet all four criteria for authorship, and all who meet the four criteria should be identified as authors. For more information on authorship, please see http://www.icmje.org/recommendations/browse/roles-and-responsibilities/defining-the-role-of-authors-and-contributors.html#two.

All listed authors meet the ICMJE criteria.  We attest that all authors contributed significantly to the creation of this manuscript, each having fulfilled criteria as established by the ICMJE.

One or more listed authors do(es) not meet the ICMJE criteria.

We believe these individuals should be listed as authors because:

[Please elaborate below]  

We confirm that the manuscript has been read and approved by all named authors.

We confirm that the order of authors listed in the manuscript has been approved by all named authors.

## Contact with the editorial office

The Corresponding Author declared on the title page of the manuscript is:

[Insert name below].

SAM S. DAHR.

This author submitted this manuscript using his/her account in EVISE.

We understand that this Corresponding Author is the sole contact for the Editorial process (including EVISE and direct communications with the office). He/she is responsible for communicating with the other authors about progress, submissions of revisions and final approval of proofs.

We confirm that the email address shown below is accessible by the Corresponding Author, is the address to which Corresponding Author's EVISE account is linked, and has been configured to accept email from the editorial office of American Journal of Ophthalmology Case Reports:

[Insert email address you wish to use for communication with the journal here].

samdahr@yahoo.com.

Someone other than the Corresponding Author declared above submitted this manuscript from his/her account in EVISE:

[Insert name below] KRISTEN-COLLISTER@DMEI.ORG.

We understand that this author is the sole contact for the Editorial process (including EVISE and direct communications with the office). He/she is responsible for communicating with the other authors, including the Corresponding Author, about progress, submissions of revisions and final approval of proofs.

We the undersigned agree with all of the above.

## CRediT authorship contribution statement

**Kristen Collister:** Writing – original draft, Writing – review & editing, Visualization. **Sam S. Dahr:** Supervision, Writing – review & editingWriting-Reviewing and Editing.

## Declaration of competing interest

No conflict of interest exists.
